# Neural responses to proxemic distance regulation in urban public spaces in the post-COVID context using a portable electroencephalogram

**DOI:** 10.3389/fpsyg.2026.1670088

**Published:** 2026-04-22

**Authors:** Shabnam Salehi, Roozbeh Naghshineh, Ali Ahmadian

**Affiliations:** 1 Islamic Azad University, West Tehran Branch, Tehran, Iran; 2 University of California Los Angeles, Los Angeles, CA, United States; 3 Shahid Beheshti University, Tehran, Iran

**Keywords:** cognitive science, COVID-19, electroencephalography, interpersonal distance, portable EEG, post-COVID, proxemics

## Abstract

Recent advances in cognitive neuroscience are increasingly informing architectural and urban studies by providing objective insights into human–environment interactions. Proxemic relationships play a critical role in shaping urban spaces, and the widespread implementation of social distancing measures during the COVID-19 pandemic may have influenced how individuals perceive and regulate interpersonal distance. This exploratory study investigates neural responses associated with proxemic distances in urban settings during the post-COVID period, referencing established proxemic frameworks rather than relying on direct pre-pandemic neural comparisons. Using a quasi-experimental design, primary data were collected through portable electroencephalography (EEG) recordings and questionnaires. Participants were exposed to controlled interpersonal distance scenarios in an outdoor urban environment, allowing examination of neural activity at personal and social distances. EEG analyses focused on alpha (8–12 Hz) and low-beta (12–15 Hz) frequency bands, which are commonly associated with relaxation and attentional engagement, respectively. Results indicate a consistent decrease in alpha power and a concurrent increase in low-beta activity when interpersonal distance was reduced, suggesting heightened vigilance and cognitive engagement in response to proxemic intrusion. Behaviorally, a subset of participants maintained interpersonal distances exceeding classical proxemic norms, highlighting variability in post-COVID distancing tendencies. These findings suggest deviations from established proxemic standards that warrant further investigation. The findings offer preliminary neurophysiological evidence relevant to urban and architectural design, emphasizing the importance of considering interpersonal distance as a factor influencing mental comfort in public spaces. Future research employing longitudinal designs, larger samples, and complementary qualitative measures is needed to clarify the persistence and origins of observed proxemic adaptations.

## 1 Introduction

In recent years, advances in cognitive neuroscience have increasingly informed architectural and urban studies by offering objective methods for examining human–environment interactions (Papale et al., 2016; Mallgrave, 2015; Proietti and Gepshtein, 2024). Traditional approaches in architecture and urban design have largely relied on behavioral observations, phenomenological interpretations, or post-occupancy evaluations to understand how individuals experience space (Rapoport, 2016). While valuable, such approaches often lack direct insight into the underlying cognitive and neurophysiological processes that shape spatial perception and behavior. The integration of neuroscience-based methodologies provides new opportunities to bridge this gap by linking environmental conditions to measurable neural responses (Abbas et al., 2024).

One critical dimension of human–environment interaction is proxemics, which refers to how individuals perceive, regulate, and negotiate interpersonal distance in social contexts (Hall, 1963; Hall, 1969). According to classical proxemic theory, interpersonal distances are commonly categorized into four zones: intimate, personal, social, and public. The present study focuses specifically on the personal and social distance ranges (Rapoport, 2016; Aiello, 1977). Classical proxemic frameworks, most notably those proposed by Edward T. Hall, describe distinct interpersonal distance zones that have been widely used in environmental psychology, architecture, and urban design (Watson and Graves, 1966). However, these frameworks have also been shown to be flexible, shaped by cultural norms, situational factors, and individual differences (Esparza-Ros and Vaquero-Cristóbal, 2025).

The COVID-19 pandemic introduced unprecedented changes to everyday spatial behavior through prolonged social distancing policies, mask mandates, and restrictions on physical proximity (Mehta, 2020; Shafiee et al., 2024). These measures altered how individuals navigated public spaces and interacted with others, potentially reshaping proxemic expectations and comfort thresholds. While a growing body of research has examined behavioral and perceptual changes associated with COVID-19, particularly in relation to public space use and interpersonal distancing (Mehta, 2020; Honey-Rosés et al., 2021; Sharifi and Khavarian-Garmsir, 2020; Ugolini et al., 2020), recent research indicates that, while actual interpersonal distances may not change substantially following the onset of the pandemic, individuals’ perception of interpersonal space and preferred distancing have been significantly altered by subjective risk and anxiety associated with COVID-19 (Givon-Benjio et al., 2024). Longitudinal and cross-sample evidence further suggests that personal space boundaries expanded during the COVID-19 pandemic, a pattern linked to social distancing practices and perceived infection risk, and observable across both real and virtual interaction contexts (Holt et al., 2022); in addition, large-scale cross-cultural studies report a global increase in preferred interpersonal distance during the pandemic period, indicating widespread behavioral adaptation of proxemic norms across different societies (Croy et al., 2024).

Recent work in environmental psychology and cognitive neuroscience suggests that intrusions into personal or social space can elicit measurable neural responses associated with vigilance, stress, and attentional engagement (Vigni et al., 2024). Electroencephalography (EEG) studies have demonstrated that reductions in interpersonal distance may be accompanied by changes in specific frequency bands, such as decreases in alpha power and increases in beta activity, which are commonly linked to heightened cognitive and emotional processing (Nolte et al., 2025). These findings highlight the potential of EEG as a tool for examining proxemic experience beyond self-report or behavioral observation alone (Taherysayah et al., 2024).

The present study builds on this interdisciplinary foundation by employing portable EEG to explore neural responses to proxemic distance regulation in an outdoor urban setting in the post-COVID context. Data were collected in 2022, a period characterized by transitional social practices following the COVID-19 pandemic. This study adopts an exploratory approach, drawing on established proxemic frameworks to contextualize observed neural and behavioral responses.

By combining neurophysiological measures with controlled proxemic scenarios, this research seeks to contribute preliminary evidence on how interpersonal distance is processed at the neural level in contemporary urban contexts. The findings aim to inform ongoing discussions in architecture and urban design regarding mental comfort, spatial configuration, and the role of interpersonal distance in shaping public space experience.

### 1.1 Aim and objectives

The main aim of the present study is to explore neural responses associated with interpersonal (proxemic) distance regulation in urban environments during the late phase of the COVID-19 pandemic, using portable (EEG) as an objective measurement tool.

To achieve this aim, the study pursues the following objectives:

1 To examine neurophysiological responses associated with different proxemic distances (personal and social) in an outdoor urban setting using portable EEG.
2 To analyze changes in alpha and low-beta EEG activity during controlled proxemic intrusions as indicators of cognitive and attentional engagement.
3 To contextualize observed neural and behavioral responses within established proxemic frameworks and discuss their potential implications for mental comfort in urban public spaces.

### 1.2 Proxemics

Proxemics refers to the field of study that examines how individuals use and regulate interpersonal distance in social interactions and how these distances are shaped by cultural, environmental, and individual factors (Esparza-Ros and Vaquero-Cristóbal, 2025; Mueller et al., 2014). The concept was first introduced by Edward T. Hall to describe the human management of space and the unwritten rules governing spatial behavior in everyday interactions (Hall, 1963, 1969).

**Figure 1:**
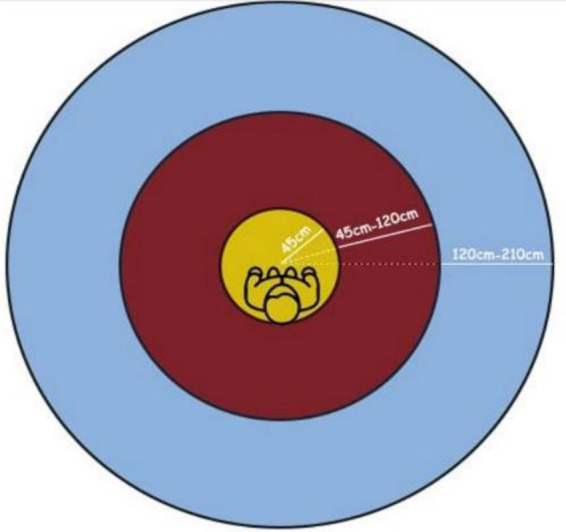
Proxemic distances.

**Figure 2:**
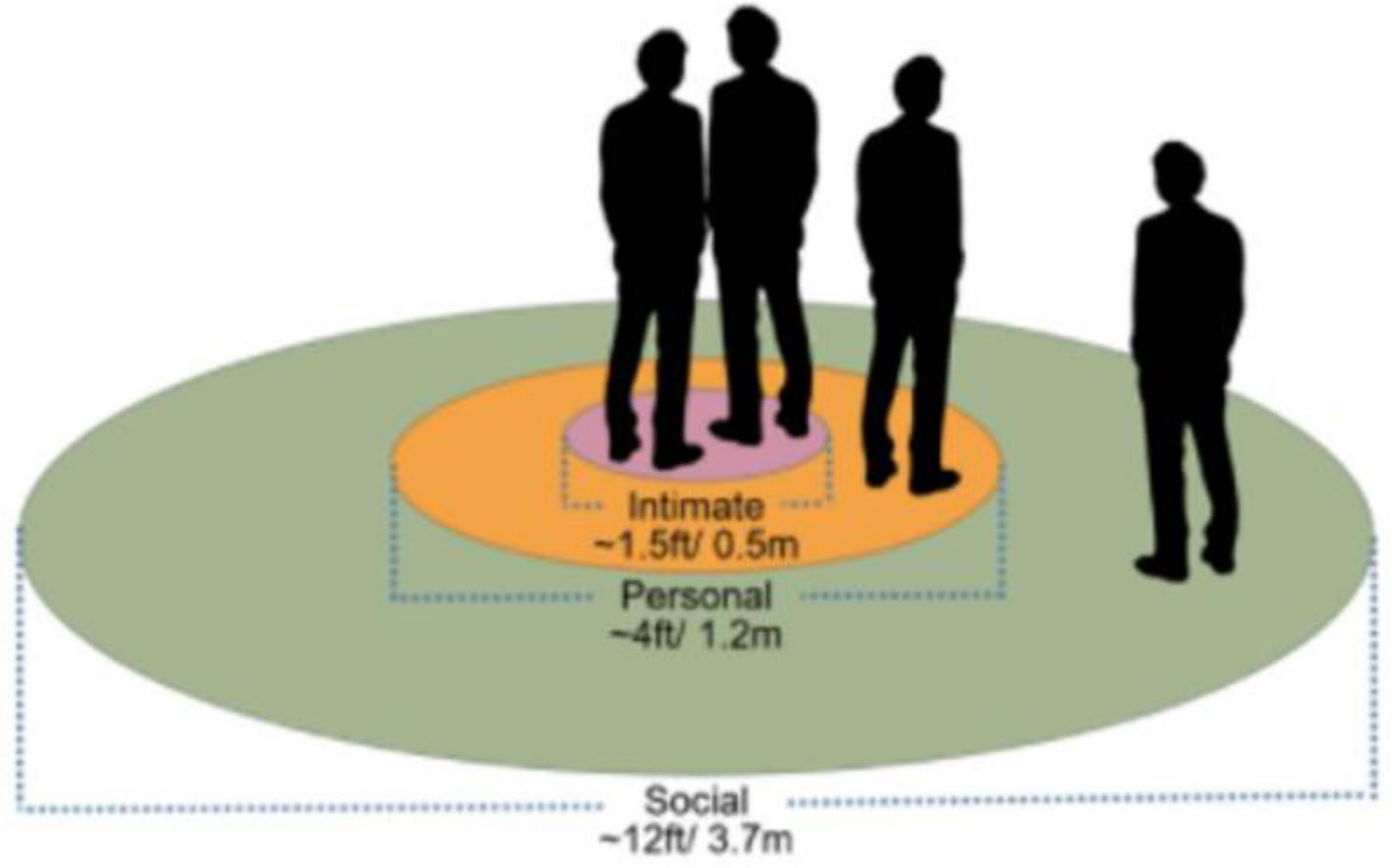
Proxemic distances (Marquardt and Greenberg, 2015).

According to Hall’s theory, interpersonal behavior is organized into four primary proxemic zones: intimate, personal, social, and public. Each zone is associated with different levels of comfort, interaction intensity, and perceptual sensitivity and extends beyond mere physical distance to include psychophysiological cues such as visual perception, voice intensity, bodily contact, and movement. Subsequent studies have demonstrated that these zones are not fixed but vary according to cultural norms, demographic characteristics, and individual personality traits (Aiello, 1977; Jones and Aiello, 1973) (Figures 1, 2).

From the perspective of environmental psychology, research indicates that when individuals perceive their surroundings as crowded or overstimulating, cognitive load and psychological stress increase. In response, people often adjust interpersonal distances as a regulatory mechanism to manage discomfort and mental strain (Evans and McCoy, 1998). In this sense, proxemic preferences function as adaptive strategies that help individuals maintain a sense of control and psychological comfort within social environments.

Recent advances in cognitive neuroscience, particularly studies employing EEG, have provided converging neurophysiological evidence for the regulatory role of interpersonal distance; experimental findings indicate that intrusions into personal space elicit measurable changes in brain activity, most notably reductions in alpha power and increases in beta-band activity, which are commonly associated with heightened vigilance, attentional engagement, and perceived threat (Llobera et al., 2010; Nolte et al., 2025). More recent EEG studies further demonstrate that alpha-band suppression occurs as interpersonal distance decreases, reflecting increased social attention and arousal during close-proximity interactions (Veranic et al., 2025; Vigni et al., 2024). Collectively, these findings suggest that proxemic behavior is not merely a social convention but is grounded in measurable cognitive and neural processes that dynamically respond to spatial proximity.

Cross-cultural research indicates that although interpersonal distance preferences vary across societies, the regulation of personal and social space constitutes a fundamental social behavior that is strongly shaped by cultural norms (Sorokowska et al., 2017). Within this framework, urban studies conducted in Iran further confirm the importance of cultural and environmental context in shaping proxemic behavior. Salehi and Naghshineh (2021) examined interpersonal distances in Iranian urban spaces prior to the COVID-19 pandemic and reported values consistent with Hall’s proxemic classification. Together, these findings provide a valuable pre-pandemic baseline for assessing potential changes in interpersonal distance regulation in the post-COVID context.

Given that proxemic behavior emerges from the interaction of cultural norms, psychological mechanisms, and neural processes, investigating potential changes following COVID-19 social distancing policies requires an interdisciplinary approach. Accordingly, the use of cognitive science tools such as EEG can contribute to a deeper understanding of neural responses to social and personal distance regulation, while also offering objective evidence to inform the redesign of urban spaces and built-environment elements in response to evolving proxemic norms.

### 1.3 Cognitive science in architecture

Cognitive science is an interdisciplinary field concerned with the scientific study of the mind and intelligence, encompassing disciplines such as psychology, neuroscience, artificial intelligence, linguistics, philosophy, and anthropology (Thagard, 2013; Nasri et al., 2024; Panwar et al., 2024). Within this framework, neuroscience focuses on the structure and function of the nervous system and seeks to explain the biological foundations of perception, cognition, behavior, and consciousness (Rose and Abi-Rached, 2013).

Understanding how the brain processes sensory and spatial information has been described as a central challenge of contemporary biological sciences (Ogawa and Oka, 2013).

Although architecture and neuroscience were traditionally regarded as separate disciplines, advances in neuroscience have increasingly demonstrated how humans perceive, interpret, and respond to the built environment. In particular, insights into perceptual and cognitive processes have shown that spatial characteristics such as layout, lighting, scale, and acoustics can influence stress levels, attentional states, emotional responses, and overall well-being (Sternberg and Wilson, 2006; Taherysayah et al., 2024). From this perspective, architectural space is not merely a physical container but an active component that shapes human experience through perceptual and cognitive mechanisms.

This reciprocal relationship has led to the emergence of neuroarchitecture, an evidence-based approach that integrates neuroscientific principles into architectural and urban design. Neuroarchitecture emphasizes the use of physiological and neural indicators, such as brain activity patterns, stress-related responses, and attentional markers, to evaluate how design elements affect human cognition and emotion (Zeisel, 2006; Abbas et al., 2024). Rather than relying solely on esthetic or functional criteria, this approach seeks to ground design decisions in measurable human responses to space.

Importantly, research in neuroscience suggests that a substantial portion of environmental processing occurs at a subconscious level, meaning that individuals may be affected by spatial conditions without explicit awareness (De Paiva, 2018). This insight is particularly relevant for understanding social interactions in space, where subtle environmental cues can influence behavior, comfort, and stress. Consequently, cognitive science approaches provide a suitable framework for examining how interpersonal distance and spatial proximity are perceived and regulated within built environments. By employing tools such as EEG, it becomes possible to investigate the neural correlates of spatial interaction and to better understand how social distances in architectural and urban contexts are cognitively and neurologically processed.

## 2 Materials and methods

### 2.1 Study design and conceptual framework

The present study employed a quasi-experimental, two-phase design conducted in an outdoor urban environment. In Phase 1, a questionnaire-based screening procedure was used to assess participants’ interpersonal communication-related skills and to select a homogeneous subgroup for experimental testing. In Phase 2, an EEG-based experiment was conducted to examine neural responses to controlled manipulations of interpersonal distance.

The experimental logic was grounded in proxemic theory, in which interpersonal distance functions as an environmental and social stimulus. In this framework, systematic manipulations of proxemic distance (personal versus social) were treated as experimental conditions, while neurophysiological responses measured via EEG constituted the primary outcomes. Conceptually, the study assumes that changes in proxemic distance elicit modulations in neural activity, particularly within frequency bands associated with attentional engagement and cognitive processing. Accordingly, the conceptual framework of the study can be described as follows: manipulation of proxemic distance leads to measurable neural responses in EEG activity, which are interpreted as indicators of cognitive engagement and comfort regulation in urban space (Table 1).

**Table 1:** Result of selected subjects’ questionnaires.

Questionnaire	Sub1 (Average answers)	Sub2 (Average answers)	Sub3 (Average answers)	Sub4 (Average answers)	Sub5 (Average answers)	Sub6 (Average answers)	Sub7 (Average answers)	Sub8 (Average answers)	Sub9 (Average answers)
Q1 friendship skills	3	3	3	3	3	3	4	4	4
Q2 confidence skills	4	3	3	3	3	3	4	3	4
Q3 non-verbal communication skills	3	4	3	3	4	3	4	3	3
Q4 COVID-19	2	2	2	1	1	1	2	2	2

### 2.2 Research questions and hypotheses

Guided by an exploratory approach, the study addresses the following research question:

How are variations in interpersonal (proxemic) distance reflected in neurophysiological responses in an urban environment? Based on prior findings in environmental psychology and cognitive neuroscience, the following hypotheses were formulated:

*H1*: Reductions in interpersonal distance are expected to be associated with measurable changes in EEG activity.

*H2*: Decreasing interpersonal distance is expected to be accompanied by a reduction in alpha-band power and a concurrent increase in low-beta-band activity, reflecting heightened cognitive and attentional engagement.

### 2.3 Variables and measures

The study variables were defined as follows:

*Independent variable*: Interpersonal distance condition, operationalized as controlled proxemic distance levels, including personal distance and social distance. Dependent variables: Neurophysiological measures derived from EEG recordings, including alpha-band power (8–12 Hz) and low-beta-band power (12–15 Hz). Control variables: Participant age and gender, environmental conditions of the experimental setting, time of day, and the fixed sequence of experimental scenarios.

*Phase 1 screening outcomes*: Scores derived from questionnaire-based assessments of friendship skills, confidence skills, and non-verbal communication abilities (Q1–Q3), as well as COVID-related perception items (Q4). These measures were used solely for participant screening and descriptive purposes and were not included as outcome variables in the EEG analysis.

### 2.4 Participants

A total of 20 participants (aged 20–30 years, mean age = 23), all physically and mentally healthy undergraduate or graduate students, completed the questionnaire-based screening in Phase 1. Based on the screening results, nine participants (five women and four men) demonstrating moderate-to-strong interpersonal communication skills were selected for Phase 2 of the study.

Prior to participation, all selected participants were screened by a physician to confirm the absence of major physical or mental health conditions. Participants reported no substance dependence. Written informed consent was obtained from all participants in accordance with the guidelines of the Iran National Committee for Ethics in Biomedical Research.

### 2.5 Instruments

#### 2.5.1 Subject selection part 1

To investigate proxemic distance regulation in urban environments during the late phase of the COVID-19 pandemic (post-pandemic context), this study followed a quasi-experimental design in two phases. In Phase 1, we developed three original questionnaires to assess friendship skills (Abbas et al., 2024), confidence skills (Aiello, 1977), and non-verbal communication abilities (Croy et al., 2024).

These instruments were developed based on a brief literature review, refined via expert feedback, and pilot-tested with a small sample (*n* = 5) to ensure item clarity. Internal consistency was assessed using Cronbach’s alpha based on the Phase 1 screening sample (*n* = 20), yielding acceptable reliability values for screening purposes (*α* = 0.72–0.81).

Preliminary reliability checks indicated acceptable internal consistency for screening purposes. The questionnaires were used exclusively for participant selection and descriptive characterization rather than inferential statistical analysis.

Preliminary checks indicated acceptable internal consistency. We also collected basic COVID-19 background data to gauge participants’ COVID-19-related perceptions.

A total of 20 participants (ages 20–30; mean age = 23), all physically and mentally healthy undergraduate or graduate students, completed the questionnaires. Responses were recorded on a 4-point scale (1 = low, 4 = high). Based on the screening results, participants demonstrating moderate-to-strong communication skills were shortlisted for Phase 2. Table 2 reports the screening scores for the nine participants who were ultimately selected for Phase 2.

**Table 2:** Screening results of the selected participants’ questionnaires.

Questionnaire	Sub1 (Average answers)	Sub2 (Average answers)	Sub3 (Average answers)	Sub4 (Average answers)	Sub5 (Average answers)	Sub6 (Average answers)	Sub7 (Average answers)	Sub8 (Average answers)	Sub9 (Average answers)
Q1 friendship skills	3	3	3	3	3	3	4	4	4
Q2 confidence skills	4	3	3	3	3	3	4	3	4
Q3 non-verbal communication skills	3	4	3	3	4	3	4	3	3
Q4 COVID-19	2	2	2	1	1	1	2	2	2

#### 2.5.2 Subject selection part 2

**Figure 3:**
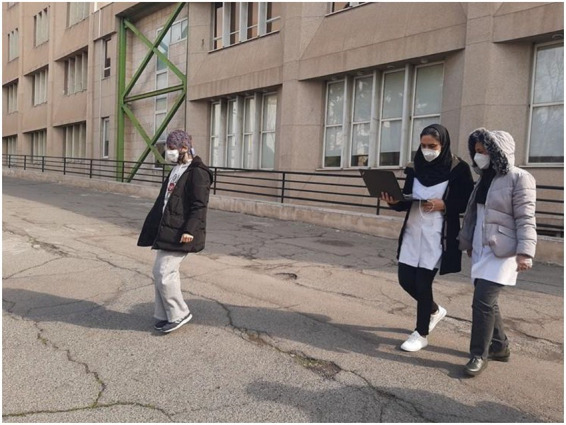
Using a portable EEG to relocate the subject to the specified location.

Following the Phase 1 screening, nine participants were selected for Phase 2 of the study. Prior to EEG data collection, a brief medical screening was conducted to confirm the absence of physical or mental conditions that could affect neural signal recording. Participants were instructed to refrain from alcohol consumption, psychoactive medication, and excessive caffeine intake within 24 h prior to the experiment and to ensure adequate sleep before participation. Before EEG data collection, participants were equipped with a portable EEG system and guided to the designated experimental location within the study site (Figure 3).

### 2.6 Experimental setting and procedure

#### 2.6.1 Setting

**Figure 4:**
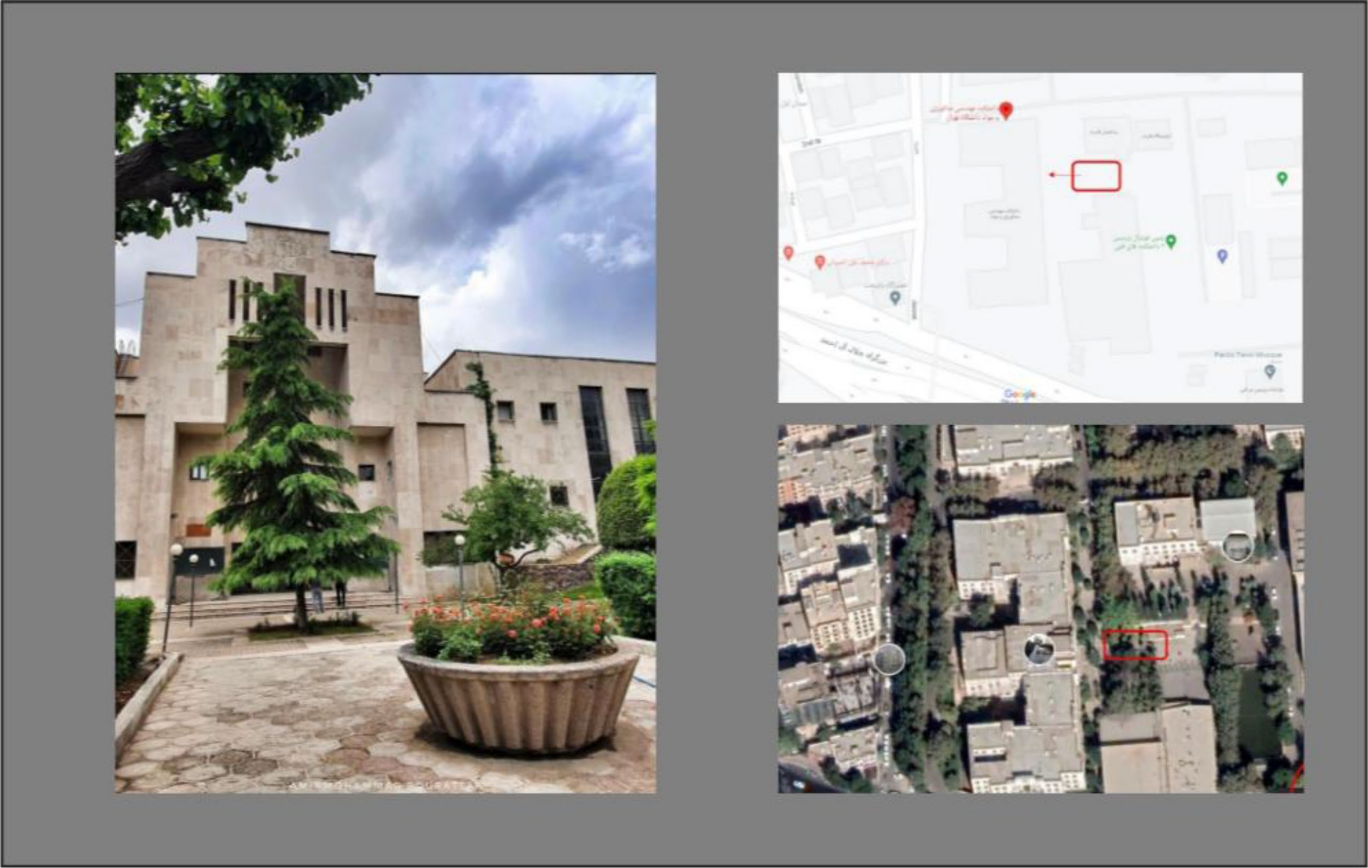
The location of the project, University of Tehran.

The experimental setting was selected based on spatial and environmental criteria aimed at minimizing external disturbances while maintaining ecological validity. The study was conducted in a semi-open urban space located on the campus of the University of Tehran, characterized by limited ambient noise, controlled pedestrian flow, and adequate visibility (Figure 4).

To operationalize proxemic distances within the experimental environment, the modular pavement pattern of the site was used as a spatial reference. The boundaries corresponding to personal and social proxemic zones were predefined and temporally marked within the EEG recording system to ensure accurate synchronization between spatial events and neural data.

#### 2.6.2 Experimental setup and scenography

**Figure 5:**
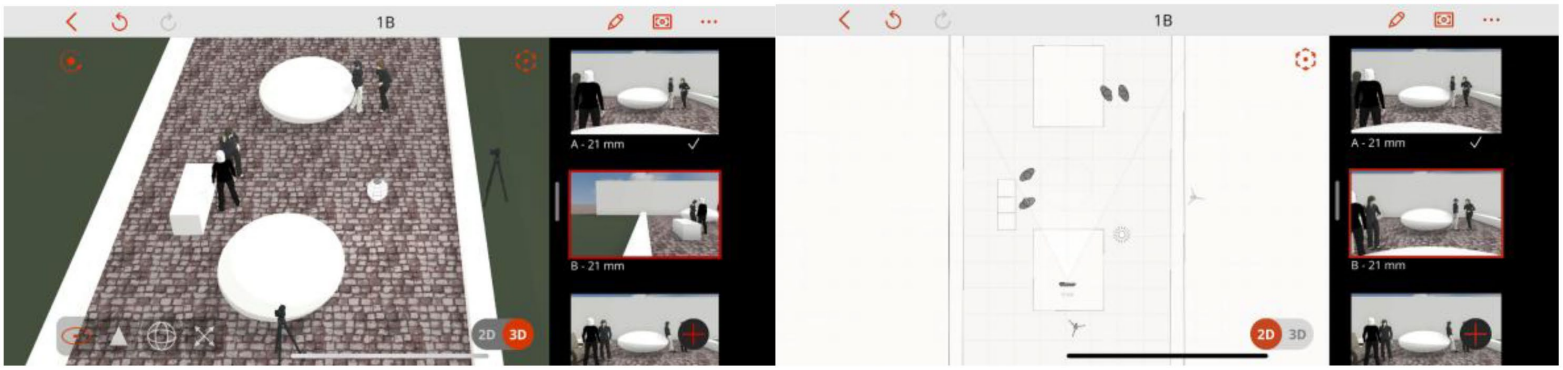
Determine the location of cameras and actors/actresses. Created by: Previs Pro App.

**Figure 6:**
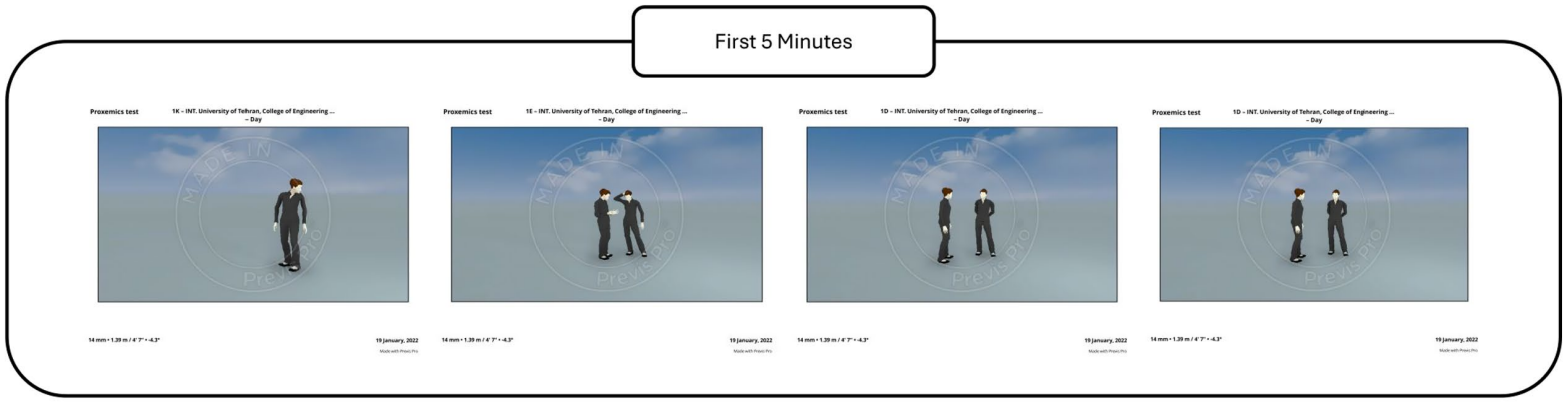
Simulation of normality, created by: Previs Pro App.

**Figure 7:**
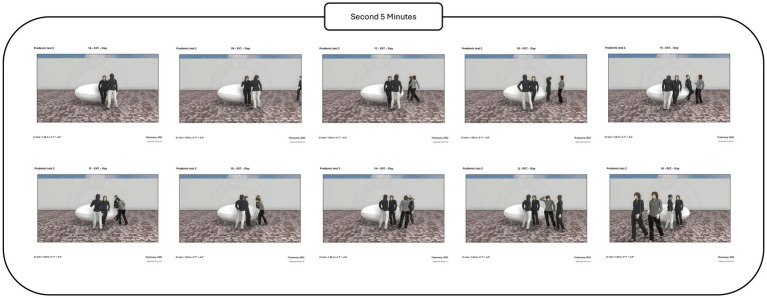
Simulation of personal distances, created by: Previs Pro App.

Prior to data collection, a pre-scenography process was conducted using the Previs Pro App to design and simulate the experimental storyboard. This process facilitated the determination of camera placement, actor positioning, and approximate lighting conditions to ensure consistency across experimental trials (Figures 5–7).

Three cameras were positioned to record the experimental scene simultaneously from different angles. Video recordings were later synchronized with EEG signals to verify the timing of key experimental events, including baseline periods and proxemic distance transitions.

### 2.7 Procedure

The experimental procedure consisted of controlled proxemic distance scenarios conducted in an outdoor urban setting. Each experimental session followed a standardized sequence to ensure consistency across participants.

**Figure 8:**
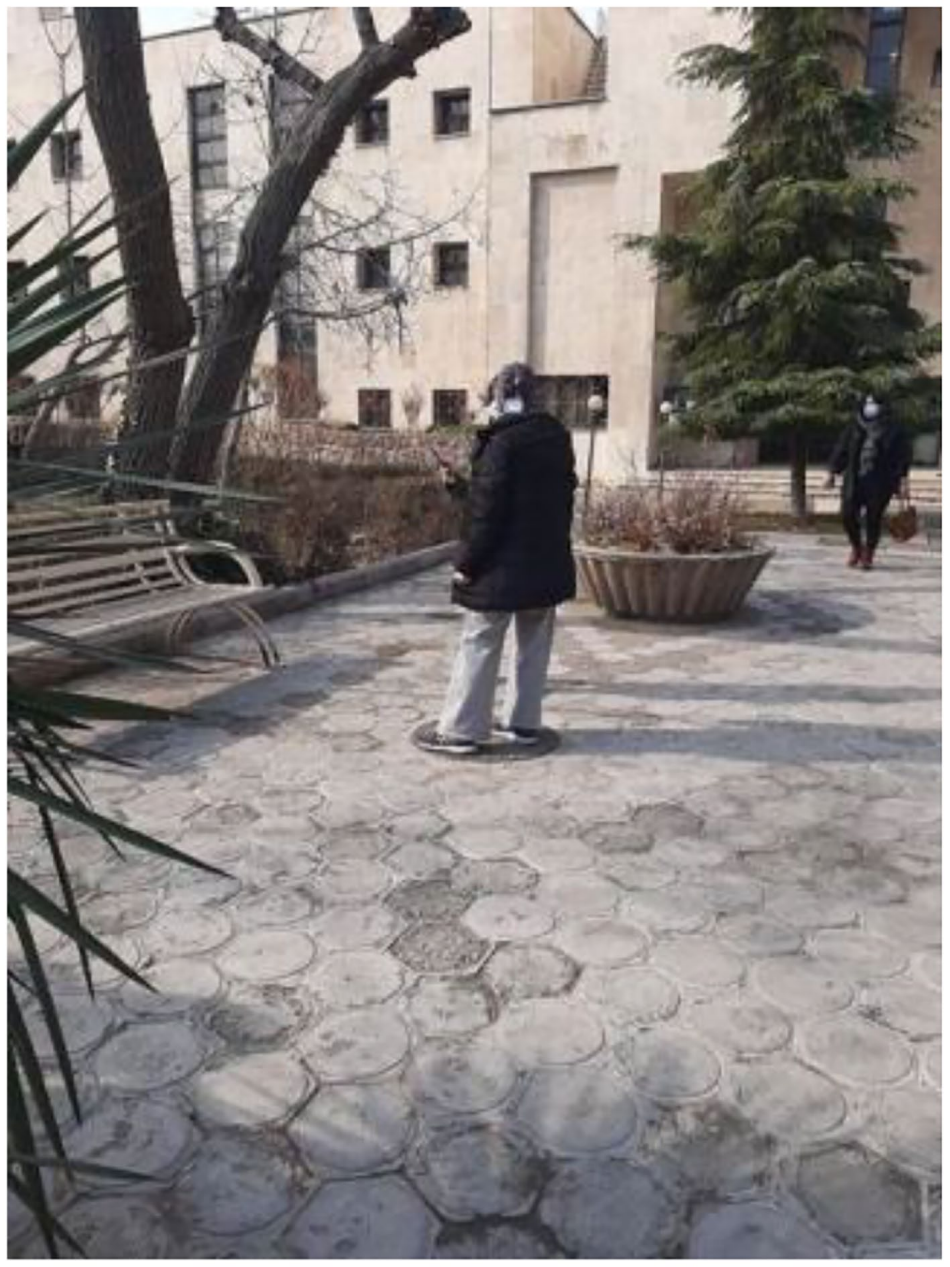
First 5-min test to analyze normality.

At the beginning of each session, a baseline period was recorded during which the participant stood alone in the designated location without any proxemic interaction. This baseline condition served as a reference state for subsequent comparisons and is referred to as the baseline condition in the analyses (Figure 8).

**Figure 9:**
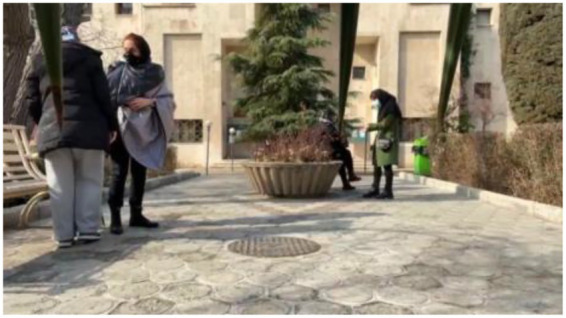
Actresses approached subjects to measure social proxemic distance.

**Figure 10:**
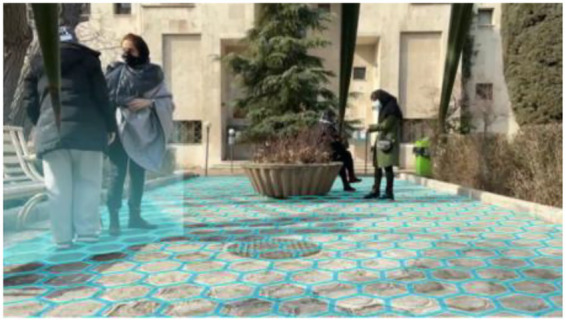
Actress approached the subject to measure personal proxemic distance.

Following the baseline period, participants were exposed to proxemic distance manipulations involving an approaching actor. The procedure included predefined proxemic conditions corresponding to social and personal distances. The actor approached the participant gradually according to the experimental script, maintaining each distance condition for a fixed duration (Figures 9, 10).

Participants were informed about the general procedure of the experiment but were not informed in advance about the specific role of the actor or the exact timing of proxemic distance changes. The experiment was conducted in 2022.

Three key temporal markers (triggers) were defined and synchronized with the EEG recording system: (Abbas et al., 2024) onset of the baseline condition, (Aiello, 1977) entry into the predefined proxemic distance zone, and (Croy et al., 2024) exit from the proxemic zone. These triggers allowed precise segmentation of EEG data corresponding to each experimental condition.

Throughout the procedure, three cameras recorded the scene simultaneously from different angles. Video recordings were later synchronized with EEG signals to verify the timing and accuracy of proxemic distance transitions and experimental events.

### 2.8 Data processing and statistical analysis

EEG data processing and statistical analyses were conducted to examine neurophysiological responses associated with different proxemic distance conditions. Raw EEG signals were visually inspected, and segments affected by excessive noise or motion artifacts were excluded from analysis. Standard preprocessing steps were applied, including band-pass filtering to isolate frequency bands of interest.

Power spectral features were extracted from the EEG signals for the alpha (8–12 Hz) and low-beta (12–15 Hz) frequency bands. These bands were selected due to their established associations with cognitive engagement, attention, and arousal. Analyses focused on predefined time windows corresponding to the baseline condition and proxemic distance conditions.

Prior to inferential analysis, the normality of EEG power distributions was assessed using the Shapiro–Wilk test. Depending on the distributional properties of the data, paired-sample t-tests or non-parametric Wilcoxon signed-rank tests were used to compare EEG activity across proxemic distance conditions. Statistical significance was evaluated at an alpha level of *p* < 0.05.

Effect sizes were calculated to estimate the magnitude of observed differences. All statistical analyses were performed using standard data analysis software.

After applying artifact removal and low-pass filtering (1–40 Hz), EEG data were analyzed across five frequency bands: alpha (8–12 Hz), beta (12–30 Hz), delta (0.5–4 Hz), gamma (30–100 Hz), and theta (4–8 Hz). The primary analytical focus was placed on low-beta (12–15 Hz), which is commonly associated with focused attention, and alpha (8–12 Hz), typically linked to mental relaxation (Konstantinidis et al., 2025).

## 3 Findings

Table 3 presents representative numerical values of relative alpha and low-beta power for Subject 6, provided as an illustrative example of the neural activity observed before and after the proxemic intrusion trigger. These data suggest relative stability in both frequency bands.

**Table 3:** Recorded data before and after trigger from 6th subject that demonstrates the changes when the actor approaches.

Band	Channel	Time (ms)										
Relative alpha		200	180	160	140	120	100	90	80	70	60	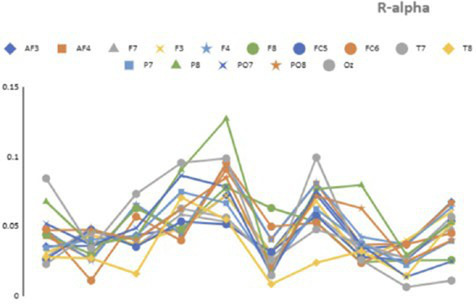
	AF3	0.0355	0.0358	0.0434	0.0482	0.0719	0.0312	0.0724	0.0338	0.0363	0.0674	
	AF4	0.0428	0.0263	0.0646	0.0441	0.0903	0.0405	0.0806	0.0365	0.0374	0.0666	
	F7	0.0278	0.0487	0.0357	0.0583	0.0531	0.0272	0.0586	0.0322	0.0279	0.0486	
	F3	0.0311	0.0434	0.0370	0.0484	0.0720	0.0310	0.0678	0.0257	0.0400	0.0602	
	F4	0.0469	0.0250	0.0655	0.0447	0.0942	0.0402	0.0810	0.0421	0.0367	0.0634	
	F8	0.0437	0.0297	0.0627	0.0477	0.0778	0.0629	0.0530	0.0240	0.0253	0.0256	
	FC5	0.0261	0.0473	0.0351	0.0532	0.0513	0.0315	0.0576	0.0278	0.0256	0.0535	
	FC6	0.0477	0.0109	0.0567	0.0397	0.0954	0.0496	0.0524	0.0235	0.0369	0.0448	
	T7	0.0227	0.0456	0.0406	0.0626	0.0561	0.0252	0.0476	0.0373	0.0265	0.0565	
	T8	0.0278	0.0268	0.0158	0.0704	0.0546	0.0083	0.0237	0.0318	0.0143	0.0522	
	P7	0.0343	0.0403	0.0430	0.0745	0.0665	0.0191	0.0622	0.0375	0.0219	0.0393	
	P8	0.0676	0.0370	0.0420	0.0903	0.1270	0.0173	0.0765	0.0794	0.0291	0.0525	
	PO7	0.0517	0.0363	0.0485	0.0863	0.0781	0.0153	0.0754	0.0365	0.0134	0.0246	
	PO8	0.0470	0.0472	0.0399	0.0621	0.0849	0.0201	0.0716	0.0627	0.0245	0.0397	
	Oz	0.0842	0.0341	0.0731	0.0954	0.0985	0.0146	0.0992	0.0256	0.0062	0.0109	
Relative low beta	AF3	0.0617	0.0825	0.0671	0.1570	0.0388	0.0673	0.1119	0.0912	0.0303	0.0859	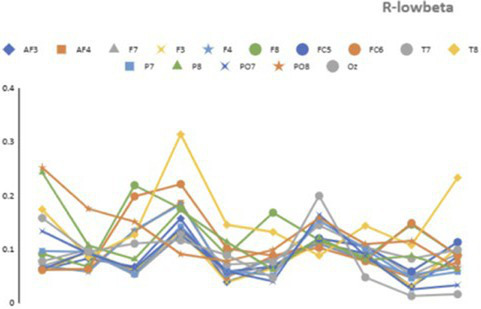
	AF4	0.0626	0.0623	0.1333	0.1861	0.0476	0.0869	0.1113	0.0769	0.0462	0.0760	
	F7	0.0687	0.0931	0.0520	0.1268	0.0533	0.0685	0.1191	0.1069	0.0516	0.0911	
	F3	0.0581	0.0967	0.0593	0.1353	0.0382	0.0644	0.1120	0.0836	0.0307	0.1031	
	F4	0.0691	0.0578	0.1350	0.1836	0.0509	0.0819	0.1097	0.0808	0.0513	0.0664	
	F8	0.0911	0.0670	0.2189	0.1755	0.0885	0.1679	0.1168	0.0846	0.1455	0.0888	
	FC5	0.0622	0.0957	0.0556	0.1268	0.0585	0.0674	0.1198	0.1049	0.0582	0.1129	
	FC6	0.0612	0.0627	0.1983	0.2211	0.1012	0.0878	0.1024	0.0775	0.1482	0.0881	
	T7	0.0773	0.0991	0.0537	0.1295	0.0712	0.0758	0.1443	0.1035	0.0822	0.0988	
	T8	0.1739	0.0852	0.1263	0.3137	0.1452	0.1318	0.0879	0.1434	0.1073	0.2331	
	P7	0.0965	0.0948	0.0530	0.1422	0.0596	0.0512	0.1533	0.0997	0.0452	0.0576	
	P8	0.2429	0.1068	0.0812	0.1726	0.1132	0.0617	0.1211	0.0791	0.0874	0.0619	
	PO7	0.1332	0.0917	0.0634	0.1379	0.0588	0.0397	0.1638	0.0877	0.0251	0.0329	
	PO8	0.2519	0.1752	0.1512	0.0906	0.0776	0.0984	0.1582	0.1093	0.1151	0.0644	
	Oz	0.1579	0.0952	0.1102	0.1161	0.0886	0.0465	0.1993	0.0474	0.0126	0.0159	

prior to the intrusion, followed by a marked shift after the actor’s approach. Figures 11, 12 visualize topographical EEG data, providing spatial distribution maps of alpha and low-beta power before and after the intrusion.

**Figure 11:**
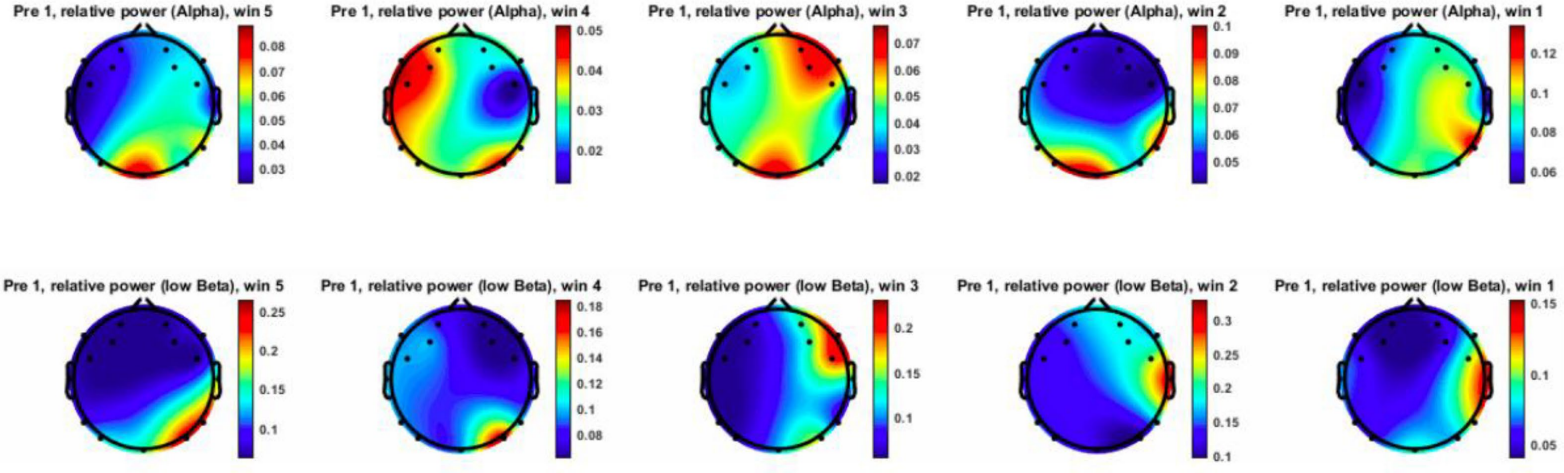
Recorded EEG data (alpha and low-beta) before the trigger for Subject 6 (pre-trigger window = win).

**Figure 12:**
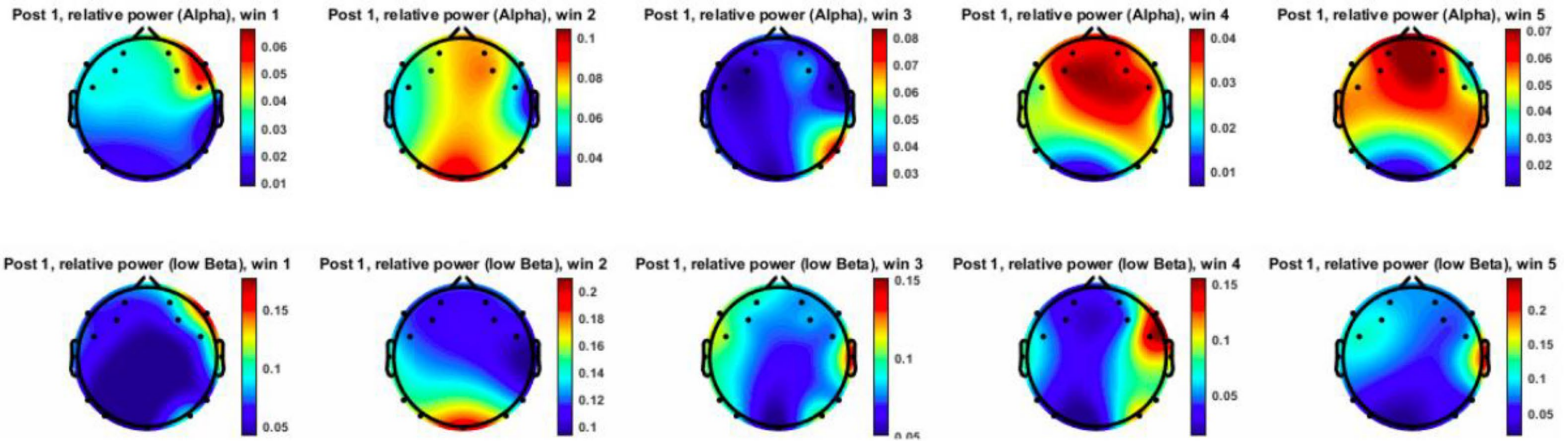
Recorded EEG data (alpha and low-beta) for Subject 6 after the trigger (post-trigger window = win).

Prior to the intrusion, alpha power was relatively high and evenly distributed, particularly in the posterior and occipital regions, indicating a comparatively relaxed neural state. Low-beta power remained moderate, reflecting a baseline attentional condition without pronounced cognitive or emotional arousal. Following the proxemic intrusion, alpha power showed a noticeable decrease, particularly in the parietal and occipital regions. This pattern is consistent with previous findings suggesting that reduced alpha activity is associated with increased vigilance and stress-related processing (Llobera et al., 2010; Vigni et al., 2024).

At the same time, low-beta power increased substantially, especially in the frontal and central regions, indicating heightened attentional processing and increased cognitive engagement in response to the actor’s approach. Together, these changes suggest an anticipatory neural response to perceived spatial boundary violations, aligning with threat-response and vigilance models proposed in proxemic interaction research.

Across participants, four out of nine individuals exhibited a noticeable increase in low-beta activity prior to the intrusion trigger, supporting the notion that proximity intrusions may elicit a preparatory cognitive response. In parallel, most participants demonstrated a reduction in alpha power, providing preliminary support for the hypothesis that proxemic intrusion is associated with a neurophysiological shift away from relaxation toward mild stress or heightened vigilance (Nolte et al., 2025).

In terms of behavioral responses, Table 4 indicates that 44% of participants maintained interpersonal distances exceeding Hall’s conventional social zone. This observation suggests that pandemic-related social distancing practices may have recalibrated participants’ perceived proxemic boundaries. The convergence of neural patterns, characterized by reduced alpha and increased low-beta activity, with behavioral distancing tendencies provides preliminary evidence that personal-space regulation in urban environments has a measurable neural component.

**Table 4:** Final results have shown 44% of participants have reacted in larger distances than the previous default distances.

Subject	Less than hall distance	Hall distance	More than hall distance	Unclassified
Subject 1		x		
Subject 2	x			
Subject 3			x	
Subject 4		x		
Subject 5				x
Subject 6			x	
Subject 7		x		
Subject 8			x	
Subject 9			x	
%	11%	33%	44%	11%

## 4 Discussion

The present findings are broadly consistent with prior research suggesting that violations of personal space can elicit measurable neural and behavioral responses (Llobera et al., 2010). In particular, the observed reduction in alpha power alongside increased low-beta activity aligns with environmental psychology studies indicating that proximity intrusions are associated with heightened vigilance or discomfort (Evans and McCoy, 1998).

Notably, the fact that nearly half of the participants maintained distances beyond Hall’s conventional social zone suggests that pandemic-related social distancing practices may have contributed to shifts in proxemic expectations. While these changes cannot be interpreted as permanent, they point to the possibility that post-COVID social norms may continue to influence interpersonal distance regulation in public spaces, with potential implications for architectural design and spatial planning.

Despite these limitations, the findings offer preliminary insights into the neural correlates of interpersonal distance regulation in urban environments. From a design perspective, the observed association between reduced alpha activity and increased low-beta power during proxemic intrusion suggests that spatial configurations minimizing involuntary close encounters may help reduce proximity-related stress. This perspective is consistent with emerging principles in neuroarchitecture, which emphasize the interaction between spatial design and cognitive-emotional responses.

Finally, several avenues for future research remain open. First, longitudinal or repeated-measures studies could help determine whether the proxemic shifts observed here persist as public health guidelines continue to relax. Second, incorporating complementary physiological measures such as heart rate variability or cortisol levels could provide a more comprehensive understanding of stress responses to personal-space violations. Third, the use of virtual or mixed reality environments may offer controlled yet ecologically valid platforms for examining whether simulated crowding elicits neural and behavioral responses comparable to those observed in real-world urban settings.

## 5 Limitations

Several limitations of the present study should be acknowledged. First, the sample size in the EEG experiment was relatively small (N = 9), which limits the generalizability of the findings and warrants cautious interpretation. Data collection was conducted during the late phase of the COVID-19 pandemic, within a broader post-COVID transitional context, a period characterized by restricted access to participants and research facilities, which constrained participant recruitment. Accordingly, future investigations with larger samples may help determine whether similar neural patterns persist under more open post-pandemic conditions.

Second, the study did not include pre-pandemic EEG measurements; therefore, direct longitudinal comparisons could not be performed. While the findings are interpreted in relation to established proxemic frameworks, further research employing repeated measurements across different temporal contexts would be valuable. In particular, should future public health crises or pandemics occur, replication of similar experimental paradigms may offer important insights into how large-scale disruptions influence proxemic regulation and neural processing over time.

Future studies employing larger and more diverse samples, multiple urban contexts, longitudinal designs, and complementary qualitative or behavioral measures are needed to further clarify the persistence and generalizability of the observed proxemic patterns.

## 6 Conclusion

This exploratory study examined neural responses associated with interpersonal distance regulation in an urban environment using portable EEG. The findings indicate that reductions in proxemic distance are accompanied by systematic changes in alpha and low-beta activity, reflecting increased cognitive engagement and vigilance during proxemic intrusion. Accordingly, the findings indicate deviations from classical proxemic expectations in contemporary urban contexts, differences whose origins and persistence warrant further investigation through future research and additional data.

By integrating neurophysiological measures with controlled proxemic scenarios, this research contributes preliminary evidence to the growing field of cognitive and neuro-informed urban studies. The findings highlight the relevance of interpersonal distance as a factor influencing mental comfort in public spaces and underscore the potential value of EEG-based approaches for studying human–environment interactions in real-world settings.

## Data Availability

The original contributions presented in the study are included in the article/supplementary material, further inquiries can be directed to the corresponding author.
